# Highlight: Museum Specimens Reveal the Secret Diversity of Bees

**DOI:** 10.1093/gbe/evaa230

**Published:** 2020-12-04

**Authors:** Casey McGrath

The past several decades have been hard on *Apis mellifera*, the Western honey bee. Originally native to Europe, Africa, and the Middle East, Western honey bees have spread worldwide thanks to the nutritional and medicinal value of their honey, pollen, royal jelly, beeswax, propolis, and venom. Even more recently, the rise of the mobile hive and increased demand for pollination services have resulted in an army of bees being unleashed on crops each year, most notably almonds, which require several million bee visits per acre. At the same time, the last 50 years have seen dramatic declines in honey bee populations due to pesticide use, climate change, and habitat destruction. Most notably, the spread of the parasitic mite *Varroa destructor* from Asia to Western Europe and North America in the 1970s decimated *A. mellifera* colonies, making it nearly impossible for honey bees to survive without human intervention and resulting in the loss of the vast majority of wild and feral honey bee colonies. Given this decline, scientists have speculated that loss of genetic diversity among honey bees may be contributing to further losses in bee populations. A new study in *Genome Biology and Evolution*, titled “Digging into the genomic past of Swiss honey bees by whole-genome sequencing museum specimens,” provides evidence that disputes this theory (Parejo et al. 2020), suggesting that loss of genetic diversity may not be among the long list of threats to bee survival.

**Fig. 1:**
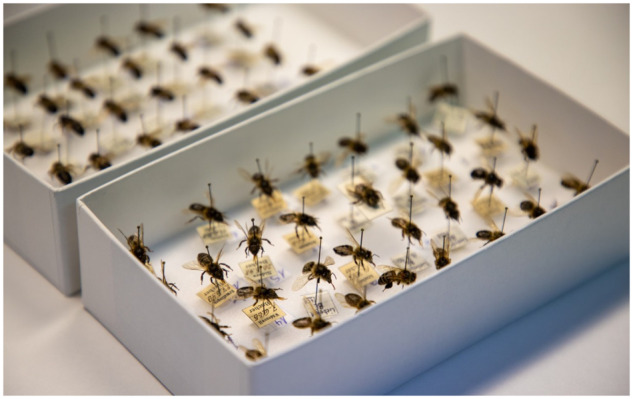
Bee specimens from the Natural History Museum in Bern, Switzerland, were sequenced to provide insight into the recent evolution of the Western honey bee. Credit: Melanie Parejo.

The study, led by Melanie Parejo, a postdoctoral researcher at the University of the Basque Country in Spain, involved the genomic sequencing of 22 bee specimens—some nearly 150 years old—from the Natural History Museum in Bern, Switzerland (fig. 1). The study represents the first whole-genome analysis of museum bee specimens, an accomplishment made possible by recent advances in high-throughput sequencing that overcome the challenges of working with historic DNA, which is often highly fragmented. By comparing the genome sequences of the historic bee samples to those of modern bees collected across Switzerland, the authors sought to uncover how changes in agricultural practices over the last 50 years had influenced the evolution of the Western honey bee.

Due to recent declines in wild bee populations and increased breeding efforts, the researchers expected to see a reduction in the genetic diversity of the modern bees compared to that of the historic specimens. However, Parejo et al. actually observed *higher* genetic diversity in the modern honey bees. “This finding was particularly surprising to us,” notes Parejo. “It was quite the opposite of what we expected and of the general narrative regarding honey bee diversity in the scientific literature, which points toward loss of genetic diversity as one of the many threats facing honey bees today.”

To explain their unexpected findings, the authors suggest that *A. mellifera’*s unique mating system, long-distance mating flights, or high recombination rate may help the Western honey bee maintain intrinsically high levels of variation. Moreover, the movement of hives and the introduction of bees from different regions may be promoting increased levels of genetic diversity in modern hives. Whatever the mechanism, this is good news for honey bees, as high levels of diversity have been shown to be crucial for colony fitness. Indeed, intracolony genetic diversity is associated with lower pathogen loads and a better chance of survival, perhaps owing to an enhanced ability to adapt to local environmental conditions.

The researchers also used the genomic data to identify signatures of selection between historic and modern Western honey bee populations. In modern bees, they found evidence for selection in immune-related genes, which may reflect the recent emergence or increasing prevalence of parasites and pathogens like *Varroa* and its associated viruses, the gut parasite *Nosema ceranea*, and the bacterium *Melissococcus plutonius*, which causes European foulbrood disease. Other genes with evidence for selection encoded nervous system proteins like ion channels and neurotransmitters, which are the targets of several widely used pesticides, including neonicotinoids and organochlorides. According to Parejo, these results “suggest that bees have had to adapt quickly to new challenges, particularly the increased use of chemicals in modern agriculture and beekeeping and the arrival of new diseases and parasites. These adaptations have left traces in the genomes of honey bees, allowing us to observe a small step in evolution.”

Overall, the results of the study should be reassuring to bee lovers, as they suggest that Western honey bees maintain sufficient adaptive potential to face future anthropogenic and environmental changes. The authors caution however that high levels of genetic diversity do not necessarily preclude the loss of specific locally adapted genetic variants, which may jeopardize colony survival. In fact, there is a recent trend toward focusing conservation efforts on “functional” diversity, rather than total genetic diversity. Toward this end, genomic analysis of museum specimens may be of further use, enabling the identification of beneficial genetic variants at specific loci that should be targeted for conservation.
